# Β-blockers treatment of cardiac surgery patients enhances isolation and improves phenotype of cardiosphere-derived cells

**DOI:** 10.1038/srep36774

**Published:** 2016-11-14

**Authors:** Isotta Chimenti, Francesca Pagano, Elena Cavarretta, Francesco Angelini, Mariangela Peruzzi, Antonio Barretta, Ernesto Greco, Elena De Falco, Antonino G. M. Marullo, Sebastiano Sciarretta, Giuseppe Biondi-Zoccai, Giacomo Frati

**Affiliations:** 1 Department of Medical Surgical Sciences and Biotechnology, “La Sapienza” University of Rome, Italy; 2 Department of Cardiovascular, Respiratory, Nephrological, Anesthesiological, and Geriatric Sciences, “Umberto I” Hospital, “La Sapienza” University of Rome, Italy; 3 Department of AngioCardioNeurology, IRCCS Neuromed, Pozzilli, Italy

## Abstract

Β-blockers (BB) are a primary treatment for chronic heart disease (CHD), resulting in prognostic and symptomatic benefits. Cardiac cell therapy represents a promising regenerative treatment and, for autologous cell therapy, the patients clinical history may correlate with the biology of resident progenitors and the quality of the final cell product. This study aimed at uncovering correlations between clinical records of biopsy-donor CHD patients undergoing cardiac surgery and the corresponding yield and phenotype of cardiospheres (CSs) and CS-derived cells (CDCs), which are a clinically relevant population for cell therapy, containing progenitors. We describe a statistically significant association between BB therapy and improved CSs yield and CDCs phenotype. We show that BB-CDCs have a reduced fibrotic-like CD90 + subpopulation, with reduced expression of collagen-I and increased expression of cardiac genes, compared to CDCs from non-BB donors. Moreover BB-CDCs had a distinctive microRNA expression profile, consistent with reduced fibrotic features (miR-21, miR-29a/b/c downregulation), and enhanced regenerative potential (miR-1, miR-133, miR-101 upregulation) compared to non-BB. *In vitro* adrenergic pharmacological treatments confirmed cytoprotective and anti-fibrotic effects of β1-blocker on CDCs. This study shows anti-fibrotic and pro-commitment effects of BB treatment on endogenous cardiac reparative cells, and suggests adjuvant roles of β-blockers in cell therapy applications.

Cardiovascular diseases (CVDs), encompassing myocardial infarction (MI), heart failure, and stroke, are a leading cause of mortality and morbidity worldwide, accounting for 17 million deaths per year. The increased survival of MI patients has led to nearly epidemic heart failure and chronic heart disease (CHD), which is responsible for more than 30% of cardiovascular related deaths, according to the American Heart Association. MI imposes mechanical and neurohormonal challenges on cardiac walls, with the initial compensatory hypertrophy eventually evolving into maladaptive remodeling^1^. Among the many consolidated therapeutic strategies, β-blockers (BB) represent a primary pharmacological treatment. Beta-blockers are able to reverse the neurohormonal effects of the sympathetic nervous system, ensuring prognostic and symptomatic benefits. Specifically, BB have been shown in randomized trials to prolong survival by preventing arrhythmia, improving CHD symptoms and left ventricular ejection fraction (LVEF), and controlling ventricular rate^2^.

Although the mechanisms are still largely unknown, treatment with β-blocking agents induces reverse remodeling^3^ at both molecular and organ level^4^. The functional improvement observed in patients treated with β-blockers can be directly linked to changes in myocardial gene expression, as these drugs act as inductors of a myocardial specific transcriptional program. As an example, molecular analyses on idiopathic dilated cardiomyopathy patients treated with β-blockers showed changes in the β-adrenergic receptors expression, and specific regulation of key genes mediating myocardial function in responsive patients^4^. Moreover, miRNA expression in the heart has been shown to be influenced by β-blockers in physiological^5^ and pathological models^6^,^7^, and they have been shown to regulate β-adrenergic receptor expression^8^.

Among the most innovative therapeutic strategies for CVDs proposed in recent years, cardiac progenitor cell (CPC) therapy has received extensive attention as a promising treatment from pre-clinical and early clinical evidence suggesting the potential to directly regenerate cardiac tissue and to activate endogenous repair, particularly for resident CPCs^9^,^10^,^11^,^12^. Despite strong transcriptomic similarities among the different resident CPC populations described in the literature^13^, the 3D model of cardiospheres (CSs) represents a unique niche-like *in vitro* microenvironment^14^,^15^, due to its tissue-like heterogeneity. CSs include cardiac progenitors, vascular progenitors and supporting mesenchymal cells, and display unique phenotypic, paracrine and regenerative features induced by the spontaneous 3D growth^16^,^17^,^18^,^19^. Recently CS-derived cells (CDCs) have successfully entered into cardiac cell therapy clinical trials, as a consolidated therapeutic cell product^20^,^21^. In addition, resident CPCs have been shown to be necessary for endogenous heart repair responses^22^.

As demonstrated for other adult stem cell types^23^,^24^,^25^,^26^, the medical history of the donor may affect endogenous regenerative abilities. Resident progenitors of most cardiovascular disease patients are naturally exposed *in situ* to multiple risk factors and elective drugs which are likely to modulate their biological features. This appears to represent a key issue to be considered in autologous strategies for the quality of the final cell product because cell number and regenerative potency are among the relevant factors affecting the success of cell therapy protocols^27^,^28^,^29^. C-kit positive cardiac progenitor cells (C-kit + CPCs) respond differentially to β-adrenergic signaling *in vitro* based on their differentiation stage^30^. Moreover, studies suggest a positive correlation between mechanical and pharmacological stress reduction and enhanced CPCs features. For example, niches of c-kit + CPCs are more abundant *in vivo* in the atria and apex^31^, which can be interpreted as a preference for microenvironments under lower contractile stress. In addition, mechanical unloading imposed on decompensated hearts by means of Left Ventricular Assist Device seems to be able to trigger favorable signaling leading to functional recovery of the myocardium, also through enhanced progenitor features^32^. Therefore, the impact of drug prescriptions and potential risk factors on resident cardiac progenitors requires careful investigation for developments and improvements of cardiac cell therapy protocols.

In the present study, we aimed to uncover correlations between clinical records of biopsy donor CHD patients undergoing cardiac surgery and the corresponding yield and phenotype of the clinically relevant CSs and CDCs therapeutic cell products. We detected a statistically significant positive correlation between β-blockers treatment and successful cardiospheres isolation, and describe for the first time a significant difference in the phenotypic balance between cardiac and fibrotic/mesenchymal features within the final therapeutic cell product of cardiosphere-derived cells linked to β-blockers treatment.

## Results

We collected in a database multiple anthropometric and clinical parameters of biopsy donors in order to determine possible correlations with the biological features of isolated CDCs (in terms of their quality and quantity as a cell product). The overall descriptive analysis of the population enrolled is presented in the first column of Table 1 (baseline features), Supplementary Table 1 (surgical indications and operative data), Table 2 (pre-operative echocardiographic parameters), and Table 3 (medical therapy). Patients undergoing elective major cardiac surgery were only enrolled in the study if atrial appendages could be safely harvested during the surgical procedure. Enrolled patients were 73% males, with a mean age of 73 ± 1.4, and a significant prevalence of hypertension and diabetes. Most of the patients had a preserved LVEF.

With respect to CSs isolation, we considered successful spheroid formation as a categorical parameter for the qualitative assessment of each biopsy, independent of its yield. We also considered the cumulative yield of CS-forming explant-derived cells (EDCs) per milligram of plated tissue for each biopsy as a continuous parameter. We have included the EDCs yield (from which the selective 3D cardiosphere stage is obtained) since it is a limiting step for the overall abundance of the final therapeutic cell product (i.e. CDCs)^21^. The unadjusted statistical analysis (Table 4) revealed a significant correlation between BB treatment in donor patients and: (i) the overall success in obtaining CSs from the explant (“Cardiospheres” parameter); (ii) the total cell number of CS-forming EDCs obtained from each biopsy (“Cumulative yield” parameter). The corresponding comparative analysis for the multiple parameters analyzed, according to BB treatment, is reported in Tables 1, 2 and 3, showing that the two patient populations (BB and non-BB) were homogeneous and consistent for most of the parameters.

To evaluate whether the correlation between CSs successful culture and BB treatment was independent of confounding variables, we performed a multivariable linear (versus cumulative EDCs yield; Table 5) and logistic (versus cardiospheres formation; Supplementary Table 2) regression analysis adjusting for valvular heart disease, which differed between BB and non-β-blocker (NBB) groups, emerging as a potentially confounding parameter (Table 1). This test showed that BB treatment was independently correlated only to CS formation and cumulative yield parameters.

**Figure 1:**
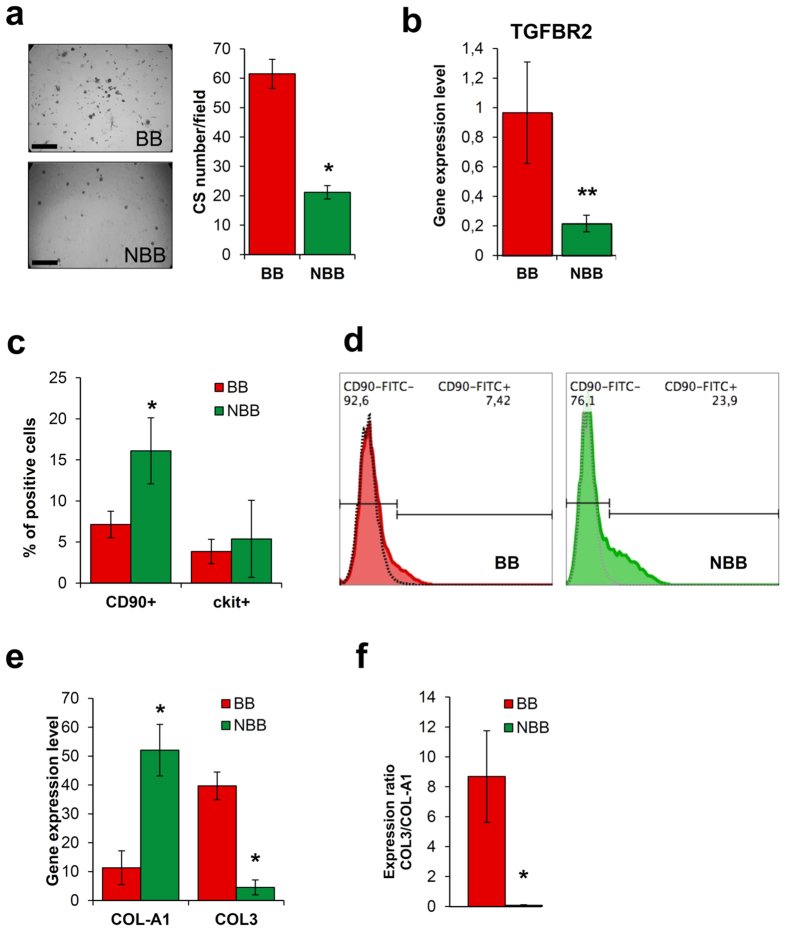
Cardiac progenitor cells phenotype differ between β-blocker and non-β-blocker patients. Representative cell culture images and corresponding CS yield quantification (**a**) show how β-blocker (BB) patients were significantly more efficient in giving CSs compared to non β-blocker (NBB) patients (when considering only the few successful NBB explants) (n = 3 biological replicates). Consistently with the CS-formation process being TGFβ-dependent, BB CPCs have significantly higher expression levels of TGFBR2 (**b**) (n = 3–7). The immunophenotypes of BB and NBB-CDCs were significantly different in the abundance of the CD90 + subpopulation (**c**) (n = 4–9), also shown by the representative flow cytometry histograms (**d**). BB and NBB CDCs also significantly differed in their expression levels of collagen I (COL-A1) and III (COL3) (**e**) (n = 3–7), and in their expression ratio (**f**). ^*^P < 0.05. ^**^P < 0.01. Scale bars = 250 μm.

Since BB treatment emerged as a strongly significant parameter in the overall success and yield of CS isolation, we investigated *ex vivo* the biological features of cells isolated from BB patients versus NBB. Β-blocker treatment, together with cellular yield, significantly affected EDCs efficiency in forming CSs. CSs yield was significantly higher for BB patients than NBB (Fig. 1a), considering only the few successful NBB explants. As the CS-formation process has been shown to be TGFβ-dependent^16^, this result was supported by significantly increased TGFBR2 gene expression in BB cells (Fig. 1b).

The overall balance of key cellular subsets inside the CDCs population was analyzed by flow cytometry and was found to be different between BB and NBB patients. While the abundance of c-kit + cells was not affected, BB patients displayed a statistically significant lower percentage of CD90 + CDCs (Fig.1c,d)^28^,^33^,^34^. These data suggest a potential reduction in CDCs prospective therapeutic efficacy, as recently described for the outcome of the CADUCEUS clinical trial^28^. In addition, the genes collagen I (COLI-A1) and III (COL3) were significantly differently expressed, with NBB-CDCs expressing higher levels of COLI-A1 and lower levels of COL3, compared to CDCs from BB patients (Fig. 1e). The COL3/COLI-A1 ratio was thus significantly reduced in NBB cells (Fig. 1f), suggesting a shift towards a more fibrotic phenotype.

**Figure 2:**
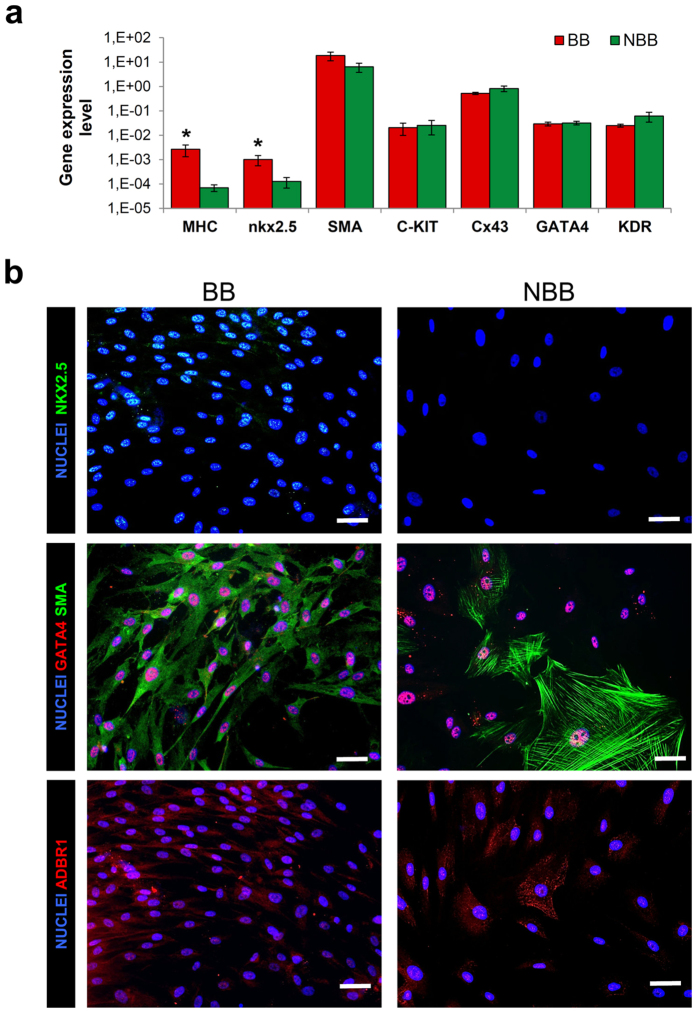
Gene expression levels and immunofluorescence phenotype of CDCs from β-blocker (BB) and non-β-blocker (NBB) patients. BB-CDCs displayed significantly higher expression levels of the cardiac specific genes MHC and Nkx2.5 (**a**) compared to NBB (NBB n = 4, BB n = 8). Representative CDC immunofluorescence images (**b**) showing a higher Nkx2.5 nuclear expression in BB cells, consistently with the qRTPCR results. Albeit no differences between BB and NBB were detectable in its expression level, intracellular staining for SMA displayed significant differences at morphological and cytoskeletal levels between BB and NBB (**b**). ADRB1 was detectable in both BB and NBB-CDCs (**b**). ^*^P < 0.05. Scale bars = 100 μm.

BB-CDCs displayed significantly higher expression of the cardiac specific genes MHC and Nkx2.5 (Fig. 2a); Nkx2.5 upregulation in cells from BB patients was also strikingly detectable at protein level by immunofluorescence (Fig. 2b) suggesting overall a more cardiac-committed phenotype. Interestingly, smooth muscle actin (SMA) expression, though not modulated at the transcriptional level (Fig. 2a), displayed a completely different cytoplasmic organization, with BB-CDCs showing an homogenous cytoplasmic distribution, whereas NBB-CDCs had a cytoskeletal organization in fibers, reminiscent of a more fibrotic or mesenchymal phenotype (Fig. 2b). No differences were detectable in the expression of GATA4 and β-adrenergic receptor 1 (ADBR1) (Fig. 2a,b). Moreover, significantly higher expression of the pro-inflammatory cytokines IL-6 and IL-8 were detectable in NBB versus BB CDCs, while other cytokines and growth factors were expressed at similar levels (Supplementary Fig. S2a).

**Figure 3:**
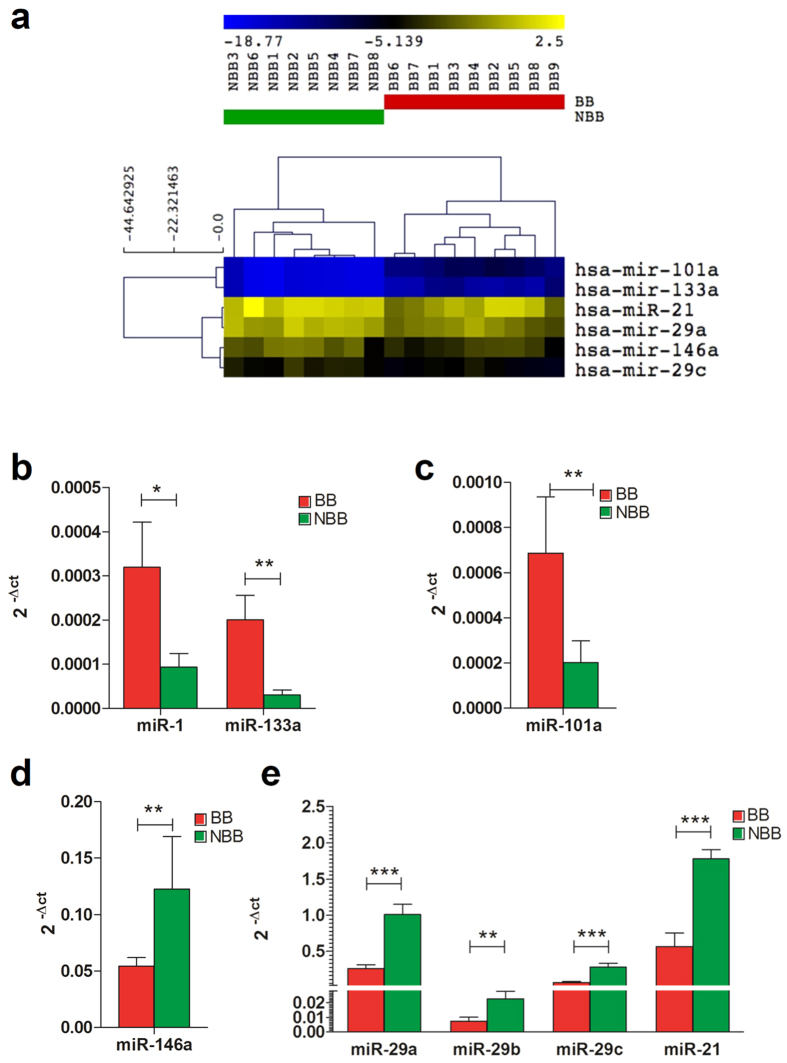
MicroRNA expression in β-blocker versus non-β-blocker CDCs. Heatmap showing hierarchical clustering analysis of miRNAs that displayed significantly different euclidean distance (**a**). Analysis was performed using averaged euclidean distance. Comparative gene expression analyses of selected miRNAs between β-blocker (BB) and non β-blocker (NBB) CDCs (**b–e**). Bar graphs show the 2^−Δct value. N = 8–9 experimental replicates, from 3 biological replicates each. ^*^P < 0.05. ^**^P < 0.01. ^***^P < 0.001.

Since microRNAs have been described to play a role in cardiac pathological fibrosis^35^,^36^,^37^,^38^,^39^,cardiomyocyte proliferation, progenitors commitment and differentiation^40^,^41^,^42^,^43^, TGF-β-mediated epithelial-to-mesenchymal transition^44^, and to correlate with CDCs therapeutic potential^45^,^46^, we selected a panel of miRNA genes to be analyzed in BB and NBB-CDCs^20^. Preference was given to those miRNAs showing altered expression due to β-blocking agents observed in other systems^6^,^7^,^47^,^48^,^49^,^50^. The relative expression of these miRNAs was quantified by realtime q-PCR. We performed hierarchical clustering analysis followed by non-parametric Kruskal-Wallis statistical test. This showed a significant difference between the two groups in terms of global expression profile of the shortlisted miRNAs. BB and NBB samples clustered separately as seen in the hierarchical dendrogram (Fig. 3a). Overall, the miRNAs related to progenitor cell survival or differentiation were significantly upregulated in CDCs from BB patients, whereas miRNAs involved in fibrosis were downregulated. Single comparisons of the expression of each miRNA in CDCs between BB and NBB patients showed that miR-1, miR-133 and miR-101 were significantly upregulated in BB patients (Fig. 3b,c), whereas miR-21, miR-29a/b/c and miR-146a were downregulated compared to NBB (Fig. 3d,e), suggesting a reduced pro-fibrotic phenotype and increased cardiac commitment in BB cells compared to NBB.

**Figure 4:**
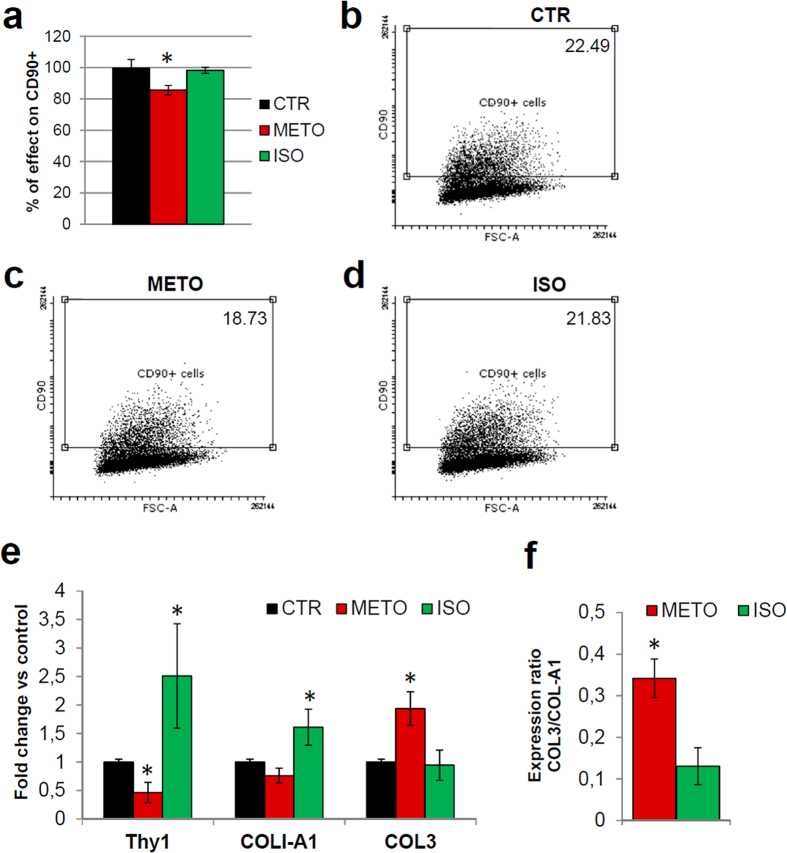
Adrenergic pharmacological treatments *in vitro* affect CDC immunophenotype and gene expression levels. The immunophenotype of BB-CDCs after 4 days of pharmacological treatment with metoprolol (METO) showed a significant reduction in CD90 + cells, expressed as percentage of effect versus control (CTR), compared to isoproterenol (ISO)-treated cells (**a**), as also shown by the representative cytofluorimetry dot plots (**b–d**). Gene expression analyses by realtime PCR consistently showed significantly lower normalized gene expression levels of Thy-1 in METO-treated cells compared to both control (CTR) and ISO-treated cells (**e**). These latter also expressed significantly higher levels of Thy1 (**e**). Moreover, METO-treated CDCs expressed significantly lower levels of collagen I (COL-A1) and higher levels of collagen III (COL3) compared to both CTR and ISO-treated cells (**e**), as also shown by their expression ratio (**f**). (n = 4). ^*^P < 0.05.

Β-blocker drugs (such as athenolol, bisoprolol, metoprolol) are preferable β1-receptor selective blockers in order to avoid the vascular and respiratory side effects of systemic β2-blockade. Therefore, in order to investigate the mechanism of BB influence on CDC abundance and phenotype, we treated short-term BB and NBB CDCs *in vitro* with 10 μM β1-receptor agonist isoproterenol (ISO) or blocker metoprolol (METO) for 4 days, either in parallel or METO after ISO (4 + 4 days). Overall, ISO-treated CDCs contained a higher percentage of early apoptotic AnnexinV + cells, but this effect was significantly recovered when CDCs were subsequently treated with METO (Supplementary Fig. S3), suggesting a cytoprotective effect of β1-blocking on CDCs. CDCs capacity of forming secondary CSs was not affected by any of the short-term *in vitro* treatments (data not shown). Interestingly, a partial but significant reduction in the CD90 + subpopulation was detectable in BB-CDCs after METO treatment compared to both untreated control and ISO (Fig. 4a–d). This is consistent with the lower percentage of CD90 cells isolated from BB versus NBB patients (Fig. 1c). Corresponding down-regulation of Thy1/CD90 gene expression levels was confirmed by realtime q-PCR (Fig. 4e). METO treatment was also able to significantly increase collagen I (COL-A1) and reduce collagen III (COL3) expression levels compared to ISO in all CDCs (Fig. 4e,f), consistent with the results from the comparative analysis between BB and NBB-CDCs (Fig. 1e,f). Finally, ISO treatment *in vitro* was associated to the same trends of significant upregulation of IL-6 and IL-8 expression levels observed in NBB-CDCs (Supplementary Fig. S2b).

## Discussion

In the scenario of autologous cardiac cell therapy clinical application, it is of great translational interest to assess how the medical background and pharmacological history of potentially eligible patients might influence the biology and the successful isolation of cardiosphere-derived cells (CDCs), currently among the most promising therapeutic cell products under clinical investigation^9^,^20^. We screened multiple medical records and CDCs *ex vivo* features for correlation discovery in order to identify predictive clinical parameters for their successful isolation and optimal regenerative phenotype, and for the possible optimization of scale-up procedures for clinical translation.

We describe for the first time a significant positive correlation between BB treatment of donor patients and both successful CSs isolation, and CS-forming cells yield from primary explant cultures (Table 4). Our results also show profound differences in cells phenotype based on their isolation from either BB or NBB patients, when considering only the minority of NBB explants that yielded CSs. In fact, a significantly faster and higher CS-forming capacity was detectable in BB explants compared to NBB (Fig. 1a). Moreover, an immunophenotypical shift of the described CDCs marker CD90^51^ was detectable between the two groups, with a significantly increased percentage of CD90 + cells in NBB (Fig. 1c). While the c-kit receptor has gradually become a highly debated cardiac progenitor cells marker^22^,^52^ and its specific role in CDCs biology and properties is negligible^27^,^34^, it has been recently reported that the expression of CD90/Thy1 is associated with a myofibroblast phenotype and lack of cardiovascular potential in CDCs^33^. Moreover, CD90 expression in injected CDCs negatively correlated with infarct scar size reduction in the CADUCEUS clinical trial^28^. Interestingly, all acute ischemic patients in the trial received β-blocker therapy (complete protocol available at: http://www.sccelltherapy.net) and the success rate in CDC isolation procedures was almost complete (only one cell manufacturing failure, mean age of ischemic donor patients of 54 years old^20^). Albeit on a significantly older cohort (mean age of 70 years old, Table 1) of chronic heart disease patients, our observations are in line with these findings, and suggest β-blocking agents prescription as an adjuvant treatment for CVD patients eligible for autologous cell therapy with resident therapeutic populations, such as CSs and CDCs. The higher percentage of CD90 + cells in NBB-CDCs could be also explained by the known proliferative effects exerted by chronic β-adrenergic stimulation on human cardiac fibroblasts^53^. To the best of our knowledge, no other surface marker has been described so far as significantly associated with influential therapeutic and biological features of the CDCs population.

Gene expression profiles revealed significantly higher collagen-IA (Fig. 1e), IL-6 and IL-8 levels (Supplementary Fig. S2), paralleled by decreased cardiac gene expression in NBB-CDCs (Fig. 2a), consistently with features of a fibroblast-enriched, and possibly more pro-inflammatory, cellular population. The expression of collagen III gene was instead decreased in NBB-CDCs, resulting in a reduced ratio of COL3/COL1 (Fig. 1f), known to be associated to fibrosis and adverse remodeling in the failing heart^54^. Interestingly, short-term *in vitro* pharmacological treatments with β1-receptor agonist (ISO) and blocker (METO) molecules mimicked the basal profiles of BB and NBB CDCs, with cells exposed to higher adrenergic drive (i.e. NBB and ISO-treated) sharing common trends of gene expression levels and CD90 + cells abundance (Fig. 4 and Supplementary Fig. 2). Moreover, METO treatment *in vitro* was associated with reduced apoptotic rate in CDCs (Supplementary Fig. 3).

Overall, our data suggest that excessive adrenergic stimulation may reduce viability of responsive cell populations, including cardiosphere-forming cells, and drive progenitors towards a fibrotic-prone and possibly pro-inflammatory phenotype. Therefore, we can speculate that biological strengthening of resident cardiac cells, with intrinsic therapeutic potential, may be another mechanism underlying the beneficial effects of clinical β-adrenergic receptors blockade, in line with results from other authors on endothelial progenitor cell and proliferating cardiogenic cells^3^,^4^. It has been reported that β1-adrenergic stimulation of committed cardiac progenitors is responsible for early myocyte precursor loss^30^. Our data show that exposure to pharmacological β-blocking may exert pro-commitment effects. The favorable outcome of β-blockers treatment in the failing heart are dependent on cardiomyocyte protection from apoptotic stimulation and, based on our findings, also on survival of an anti-fibrotic and potentially therapeutic endogenous pool. In this way β-adrenergic blockade seems to act as a pharmacological intervention able to enhance the reparative response in the failing heart.

It has been reported that cardiac progenitors are necessary for endogenous heart repair responses^22^, and supposed features of enhanced function have been described to represent positive prognostic indices for coronary bypass patients^55^. In line with this evidence, a long term follow-up is ongoing in our study cohort to clarify whether efficient CSs isolation and their phenotype may be suggested as prognostic factors in chronic heart disease patients as well.

Among the many cellular and molecular pathways involved in cardiovascular diseases onset and progression, also a deranged miRNAs expression has been observed^56^. miRNA are well described post-transcriptional regulators of gene expression, participating in many pathophysiological mechanisms in the cardiovascular system^56^, as well as in progenitors biology and cardiomyocyte proliferation^57^,^58^. Additionally, miRNA expression can be affected by β-blockers treatment, and miRNAs can be directly involved in the β-adrenergic transduction cascade^5^,^7^,^8^. A recent analysis showed that altered miRNAs expression due to MI could be reversed by β-blocker propranolol treatment in rats, suggesting that this drug can specifically revert MI-induced miRNA expression changes^6^. In our analysis, the expression profile of a selected panel of miRNAs was significantly different between the therapeutic CDCs population expanded from either BB or NBB patients. Among the miRNAs analyzed, miR-1 and miR-133a were significantly upregulated in BB-CDCs. These muscle specific myo-miRs control skeletal and cardiac muscle differentiation and survival. The expression of miR-1 can enhance embryonic stem cells differentiation into cardiomyocytes^59^, and was also shown to be upregulated upon induction of cardiac differentiation of human cardiac progenitors isolated from fetal heart^42^. MiR-133a has been shown to exert a protective anti-apoptotic effect on cardiomyocytes exposed to oxidative stress, and transplantation of miR-133a-overexpressing CDCs promoted increased functional recovery in a murine MI model^43^. Moreover, cardioprotective miR-133a expression can be induced *in vitro* by β-blocker carvedilol treatment^48^. It also protects cardiomyocytes from apoptosis induced by constitutive activation of the β-adrenergic signaling^7^, highlighting its link to BB treatment and protective cardiac mechanisms. Moreover, miR-133 was shown to negatively regulate extra-cellular matrix deposition, thus reducing interstitial fibrosis in the heart^60^. Consistently with these observations, the increase of miR-133a in CDCs collected from BB-patients was associated with features of reduced pro-fibrotic phenotype and increased cardiac commitment. Accordingly, these features were also associated with increased miR-101 expression in BB-CDCs versus NBB. In fact, miR-101 has been shown to suppress cardiac fibroblasts proliferation post-MI, with decreased collagen deposition and recovery of the impaired cardiac function^37^.

MiR-21 and the miR-29a/b/c family members are among the best characterized miRNAs involved in heart fibrosis and are highly expressed in fibroblasts^38^. Interestingly, they were all significantly downregulated in BB versus NBB-CDCs. High levels of miR-21 directly correlate with TGFβ-mediated heart fibrosis, and miR-21 was shown to be upregulated in cardiac fibroblasts after stress^35^. Our data suggest that the decreased expression of miR-21/miR-29 family observed in CDCs from BB patients might reflect the reduced proportion of pro-fibrotic cells within the BB-CDC population, which also correlated with a decreased percentage of CD90 + cells, overall suggesting a reduced fibrotic potential of BB-CDCs, which is desirable for any therapeutic cell product.

In conclusion, discovering and interpreting the connections between clinical-pharmacological features and molecular signals that may impair reparative processes or conversely improve cell survival represents a primary step for developing novel interventional therapeutic strategies aimed at enhancing myocardial healing. This study supports the possible predictive and adjuvant role of β-blocker treatment in cardiac cell therapy applications, as recently suggested for mesenchymal stem cell-based therapies^61^. It also suggests novel insights on the influence of BB treatments on the quality and abundance of the cardiac reparative cellular pool.

Besides negative inotropic action, multiple biological mechanisms have been described to be responsible for the beneficial effects of β-blockers therapy, such as antioxidant effects, increased cardiomyocyte resistance to cell death and recovery of calcium handling dynamics^48^,^62^,^63^. Our data supports a protective effect of β-blockers treatment on the endogenous cardiac reparative pool, proposing for the first time an anti-fibrotic and pro-commitment mechanism at molecular level, confirmed by *ex vivo* mechanistic assessment of the effects of β1-adrenergic signaling on CDC biology. These results lay the foundations for future improvements of CDCs isolation method, clinical assessment in cellular transplantation procedures, and for the enhancement of endogenous myocardial responses to injury mediated by β-blocking agents.

## Methods

### Study population and design

This was a prospective observational study, approved by the pertinent ethics committee of Umberto I General Hospital - Sapienza University of Rome, and performed in conformity with the declaration of Helsinki. All patients provided written informed consent. A total of 41 patients undergoing cardiac surgery at one single academic medical center were included in our analysis. The inclusion criteria were as follows: 1) Age >18 <80 years, 2) Patients undergoing on-pump major cardiac surgery. The exclusion criteria were as follows: 1) life expectancy <1 year; 2) presence of malignancies; 3) conditions precluding adherence to the protocol (7 patients excluded); 4) pregnancy; 5) enrollment in other clinical research trial; 6) patients unable or unwilling to give informed written consent; 7) BB treatment started less than 30 days before cardiac surgery or a proved non-compliance to BB therapy (2 patients excluded). A complete preoperative evaluation by a multidisciplinary team included demographic data, blood tests, 12-lead electrocardiography, and pharmacological therapy. Patients were considered under BB treatment if the drug was started at least 30 days before surgery. If no data about the length of BB treatment were available, patients were excluded from the study. In all patients a 2D Doppler echocardiography was performed as previously described^64^. LV end-diastolic and end-systolic volumes were calculated using the Teichholz method^65^.

### Cell cultures

Surgical biopsies of left atrial appendages were collected at the time of venous cannulation from patients undergoing on-pump major cardiac surgery, and cultured as explant culture. CPCs were isolated with the standardized CS protocol, as previously described^15^,^21^,^66^. Briefly, after 4 weeks of explant outgrowth, explant-derived cells (EDCs) were collected every 7 days (up to 3 times from each explant), and seeded on poly- D-lysine (BD-Biosciences) coated wells (9000 cells/cm^2^) to obtain CSs. These were then collected and expanded on fibronectin (BD-Biosciences) coated surfaces as cardiosphere-derived cells (CDCs), and expanded for not more than two split rounds, depending on the variable yield of each patient. Phenotypic as well as molecular analyses on CS and CDCs have been performed on passages number 1 or 2 (first or second harvest from the explants culture). Secondary CSs (IICSs) were obtained by replating CDCs in CS-forming conditions. CS yield (normalized to growth surface) and dimensions were calculated 1 week after plating (averaging all harvests for each biopsy) from image analysis of at least 8 random fields of culture plates, by binary transformation and automated particle count with ImageJ software (NIH). Considering the significantly lower culture success and yield of non β-blockers biopsies, the number of available biological replicates could not always match that of cells from BB donors.

### Pharmacological treatments

CDCs from four different patients were treated *in vitro* in media supplemented with 0, 1% FBS. Cells from each patient were cultured in parallel for 4 days with 10 μM isoproterenol (ISO) (Monico) or 10 μM metoprolol (METO), a selective β1-receptor blocker (AstraZeneca), using cells cultured in media alone as control group. Furthermore, another group of cells was cultured for 4 days with 10 μM ISO, and then with 10 μM metoprolol for further 4 days. Regardless of the culture timing, media were changed every 48 hours. Cells were collected after treatments and analyzed by flow cytometry and realtime qPCR, as described below, or plated from each condition on Poly-D-Lysine coated plates for CS formation assay.

### Flow cytometry analysis

Cells were grown as CDCs and the percentage of cells expressing CD90 and c-kit markers was assessed by flow cytometry. CDCs from the first explant harvest and at passage 1 were used. Confluent CDCs were harvested with trypsin-EDTA, and stained with CD90-FITC (Dianova) and CD117 (c-kit)-APC (eBioscience) antibodies, according to the manufacturer’s guidelines. For apoptosis detection, CDCs were stained with Annexin V/7-AAD kit (BD Pharmingen), according to the manufacturer’s guidelines. All acquisitions were performed using BD FACS-Aria II (BD Biosciences) and data were analyzed with FlowJo software and DiVa Software (v6.1.1).

### RNA extraction and qPCR

Total RNA was extracted using either the miRNeasy Mini or Micro Kit (Qiagen). RNA was quantified using a spectrophotometer. For gene expression analyses, cDNA was synthesized using 0.5 μg RNA, with the High Capacity cDNA Reverse Transcription Kit (Life Technologies). Real-time qPCR was performed to assess gene expression, using Power SYBR Green PCR Master Mix (Life Technologies) and standard thermocycling conditions according to the manufacturer’s protocol. For microRNA analyses, quantification was made using specific Taqman miRNA assays. Briefly 10 ng of RNA were converted in cDNA using sequence specific looped primers; each miRNA was then quantified by qPCR using specific Taqman miRNA assays and the Taqman Universal Master Mix no UNG (Life Technologies). The relative ratio versus control sample was calculated using the comparative Ct method (2^−ΔΔCt). The set of gene analyzed and the primers sequences are listed in Supplementary Table 3. GAPDH and HPRT1 (averaged Ct) were selected according to the Norm Finder software, as housekeeping genes for gene expression analyses^67^. For CDCs microRNA gene expression, U6 was used as internal control. The PCR data (delta-Ct value) were analyzed using MeV software (TM4 Software Suite) to generate the heatmap.

### Immunofluorescence

Cells were fixed for 10 minutes with 4% paraformaldehyde at 4 °C, permeabilized with 0.1% Triton X-100 (Sigma) in PBS with 1% BSA, then blocked in 10% goat serum, incubated overnight at 4 °C in 1% goat serum with primary antibodies, and then incubated for 2 hours at room temperature with Alexa-conjugated secondary antibodies (Invitrogen). Primary antibodies were: ADBR1 (Santa Cruz), αSMA (Sigma), GATA-4 and Nkx2.5 (both Abcam). Slides were mounted in ProLong Gold antifade reagent with DAPI (Life Technologies). Imaging was performed on a Nikon Eclipse Ni microscope equipped with VICO system and NIS-Elements AR 4.30.02 software.

### Statistical analysis

Descriptive analysis was based on median (1^st^ quartile, 3^rd^ quartile) for continuous variables, and count (percentage) for categorical variables, whilst bar graphs displaying mean ± standard error of the mean were used for illustrative purposes. Unadjusted inferential analysis was based on Mann-Whitney U, Kruskal-Wallis, and Spearman tests for continuous variables, and Fisher exact test for categorical variables. For microRNA analysis, clustering was performed setting average Euclidean distance and Kruskal-Wallis test to visualize significant genes (MeV software; TM4 Software Suite). Multivariable linear or logistic regression analyses were also conducted to adjust for potential confounders. Computations were performed with Stata 13 (StataCorp), SPSS 20 (IBM) and GraphPad Prism 5 (GraphPad Software).

## Additional Information

**How to cite this article**: Chimenti, I. *et al.* Β-blocker treatment of cardiac surgery patients enhances isolation and improves phenotype of cardiosphere-derived cells. *Sci. Rep.* **6**, 36774; doi: 10.1038/srep36774 (2016).

**Publisher’s note:** Springer Nature remains neutral with regard to jurisdictional claims in published maps and institutional affiliations.

## Supplementary Material

Supplementary Information

## Figures and Tables

**Table 1:** Comparative analysis of baseline features of patients, overall or according to β-blocker therapy.

Feature*	Overall	No β-blocker therapy	Β-blocker therapy	P†
Female	12/41 (29.3%)	5/19 (26.3%)	7/22 (31.8%)	0.744
Age, years	73 (64; 76)	74 (67; 76)	70 (61; 76)	0.402
Body mass index, Kg/cm^2^	27.0 (24.0; 28.4)	25.0 (23.7; 27.7)	27.5 (24.5; 28.5)	0.178
Diabetes mellitus	13/41 (31.7%)	6/19 (31.6%)	7/22 (31.8%)	1.0
Insulin-dependent diabetes mellitus	3/41 (7.3%)	0/19	3/22 (13.6%)	0.235
Hypertension	36/41 (87.8%)	17/19 (89.5%)	19/22 (86.4%)	1.0
Smoking history				0.652
Never	25/41 (60.9%)	10/19 (52.6%)	15/22 (68.2%)	
Former	12/41 (29.3%)	7/19 (36.8%)	5/22 (22.7%)	
Current	4/41 (9.7%)	2/19 (10.5%)	2/22 (9.1%)	
Atrial fibrillation	6/41 (14.6%)	3/19 (15.8%)	3/22 (13.6%)	1.0
Prior or recent acute myocardial infarction	12/41 (29.3%)	5/19 (26.3%)	7/22 (31.8%)	0.744
Recent acute myocardial infarction	8/41 (19.5%)	3/19 (15.8%)	5/22 (22.7%)	0.703
Surgical indication				
Ischemic heart disease	29/41 (70.7%)	12/19 (63.2%)	17/22 (77.3%)	0.493
Valvular heart disease	21/41 (51.2%)	14/19 (73.7%)	7/22 (31.8%)	0.012
Vascular disease	6/41 (14.6%)	2/19 (10.5%)	4/22 (18.2%)	0.668
Other	2/41 (4.9%)	2/19 (10.5%)	0/22	0.209
Heart rate, bpm	75 (64; 86)	75 (65; 86)	75 (60; 87)	0.931
Systolic blood pressure, mmHg	130 (110; 132)	130 (110; 140)	120 (110; 130)	0.322
Diastolic blood pressure, mmHg	70 (70; 80)	70 (70; 80)	80 (70; 80)	0.598
ACEF score	1.5 (1.1; 1.8)	1.4 (1.1; 1.6)	1.5 (1.3; 1.8)	0.177
EuroSCORE II	3.1 (1.9; 6.5)	3.4 (1.9; 5.3)	2.7 (2.1; 9.6)	0.356
STS score	1.9 (1.0; 3.8)	2.2 (1.4; 3.6)	1.8 (0.9; 4.7)	0.632
Blood analysis				
Glucose, mg/dl	106 (89; 121)	98 (85; 119)	110 (95; 123)	0.364
Total cholesterol, mg/dl	173 (142; 229)	187 (171; 237)	155 (131; 203)	0.123
High density lipoprotein cholesterol, mg/dl	48 (36; 62)	48 (46; 58)	40 (35; 68)	0.413
Creatinine, mg/dl	1.0 (0.9; 1.1)	1.0 (0.8; 1.1)	1.1 (0.9; 1.2)	0.371
Estimated glomerular filtration rate, mL/min/1.73 m^2^	71 (57; 88)	75 (63; 92)	71 (57; 81)	0.388
Erythrocyte sedimentation rate, mm	21 (11; 39)	25 (7; 44)	21 (19; 24)	1.0

^*^Reported as median (1^st^; 3^rd^ quartile) for continuous variables and count/total (percentage) for categorical variables.

^†^Computed with unpaired Mann-Whitney U test for continuous variables and Fisher exact test for categorical variables.

**Table 2:** Comparative analysis of pre-operative echocardiographic parameters according to β-blocker therapy.

Variable*	Overall	No β-blocker therapy	Β-blocker therapy	P†
EDD, mm	51 (49; 54)	51 (47; 55)	52 (49.2; 53.7)	0.612
ESD, mm	33 (30; 36)	33 (29.5; 35.2)	33 (30; 37)	0.525
EDV, ml	123 (112; 141)	123 (102; 147)	129 (113; 139)	0.612
ESV, ml	44 (35; 54)	44 (33; 52)	44 (35; 58)	0.525
EF, %	63.4 (60; 69)	63.6 (58.8; 71.9)	63.1 (60.4; 68.5)	0.602

EDD: end diastolic diameter. EDV: end diastolic volume. EF: ejection fraction. ESD: end systolic diameter. ESV: end systolic volume.

^*^Reported as median (1^st^; 3^rd^ quartile) for continuous variables and count/total (percentage) for categorical variables.

^†^Computed with unpaired Mann-Whitney U test for continuous variables and Fisher exact test for categorical variables.

**Table 3:** Comparative analysis of medical therapy of patients, overall or according to β-blocker therapy.

Drug*	Overall	No β-blocker therapy	Β-blocker therapy	P†
Angiotensinogen converting enzyme inhibitor	18/41 (43.9%)	9/19 (47.4%)	9/22 (40.9%)	0.758
Aldosterone receptor antagonist	8/41 (19.5%)	2/19 (10.5%)	6/22 (27.3%)	0.249
Angiotensin II receptor antagonist	12/41 (29.3%)	6/19 (31.6%)	6/22 (27.3%)	1.0
Antiarrhythmic	4/41 (9.7%)	1/19 (5.3%)	3/22 (13.6%)	0.610
Aspirin	22/41 (53.6%)	11/19 (57.9%)	11/22 (50.0%)	0.756
Calcium-channel antagonist	10/41 (24.4%)	7/19 (36.8%)	3/22 (13.6%)	0.144
Diuretic	22/41 (53.6%)	9/19 (47.4%)	13/22 (59.1%)	0.538
Statin	26/41 (63.4%)	12/19 (63.2%)	14/22 (63.6%)	1.0

^*^Reported as median (1^st^; 3^rd^ quartile) for continuous variables and count/total (percentage) for categorical variables.

^†^Computed with unpaired Mann-Whitney U test for continuous variables and Fisher exact test for categorical variables.

**Table 4:** Comparative analysis of cardiac progenitor cell isolation success and yield according to β-blocker therapy of donor patients.

Variable*	No β-blocker therapy	Β-blocker therapy	P†
Cardiospheres	7/19 (36.8%)	18/22 (81.2%)	0.005
Cumulative yield (EDCs/mg)	0 (0; 1,308)	3,319 (464; 6,012)	0.010

EDC: explant-derived cells. P1: first EDC collection.

^*^Reported as median (1^st^; 3^rd^ quartile) for continuous variables and count/total (percentage) for categorical variables.

^†^Computed with unpaired Mann-Whitney U test for continuous variables and Fisher exact test for categorical variables.

**Table 5:** Multivariable linear regression analysis for the association of EDCs cumulative yield and β-blocker therapy, adjusting for valvular disease.

Independent variable	Regression coefficient (95% confidence interval)	P
Β-blocker	3450.14 (315.52; 6584.76)	0.032
Valvular disease	440.58 (−2682.49; 3563.66)	0.775
