# Supplementary material for: Β-blockers treatment of cardiac surgery patients enhances isolation and improves phenotype of cardiosphere-derived cells
Source: Sci Rep. 2016 Nov 14;6:36774. doi: 10.1038/srep36774 (PMC5107949; doi:10.1038/srep36774)
Supplement: Supplementary Information [file srep36774-s1.pdf]

Beta-blocker treatment of cardiac surgery patients enhances isolation and improves phenotype of cardiosphere-derived cells.

Isotta Chimenti PhD[1*#], Francesca Pagano PhD[1#], Elena Cavarretta MD PhD[1], Francesco Angelini PhD[1], Mariangela Peruzzi MD PhD[1], Antonio Barretta MD[2], Ernesto Greco MD[2], Elena De Falco PhD[1], Antonino G.M. Marullo MD[1], Sebastiano Sciarretta MD PhD[1,3], Giuseppe Biondi-Zoccai MD[1,3], Giacomo Frati MD[1,3].

[1]Department of Medical Surgical Sciences and Biotechnology, “Sapienza” University of Rome, Italy.

[2]Department of Cardiovascular, Respiratory, Nephrological, Anesthesiological, and Geriatric Sciences, “Umberto I” Hospital, “Sapienza” University of Rome, Italy.

[3]Department of AngioCardioNeurology, IRCCS Neuromed, Pozzilli, Italy.

[#]Authors equally contributed.

*Corresponding author. Address for correspondence: Isotta Chimenti, Corso della Repubblica 79, 04100 Latina, Italy. Phone: +3907731757234. Fax: +3907731757254. Email: isotta.chimenti@uniroma1.it.

Abbreviations list.

CVD: cardiovascular disease

MI: myocardial infarction

CHD: chronic heart disease

BB: beta-blocker

NBB: non beta-blocker

LVEF: left ventricular ejection fraction

miRNA: miR: micro-RNA

CPCs: cardiac progenitor cells

CSs: cardiospheres

CDCs: cardiosphere-derived cells

EDCs: explant-derived cells

Supplementary Table 1. Indication for surgery and operative data.

Indication for surgery	Overall N=41	No Beta-blockers N=19	Beta-blockers N=22	p
**Mitral Valve Disease, n**	10/41 (24%)	6/19 (32%)	4 (18%)	0.469
**Aortic Valve Disease, n**	18/41 (44%)	11/19 (58%)	7/22 (32%)	0.122
**Coronary artery disease**	28/41 (68%)	11/19 (58%)	17/22 (77%)	0.313
**Coronary artery by-pass graft performed**	2.3±1	2.1±1.4	2.5±0.8	0.157
**Ascending aorta disease, n**	5/41 (12%)	1/19 (5%)	4/22 (18%)	0.350
**Associated procedures, n**	5/41 (12%)	4/19 (21%)	1/22 (4%)	0.164
**Cardio-Pulmonary By-pass Time, min**	147±63	159±72	134±49	0.283
**Cross Clamp Time, min**	113±58	125±69	99±40	0.269

Supplementary Table 2. Multivariable logistic regression analysis for the association of cardiosphere formation and beta-blocker therapy, adjusting for valvular disease.

Independent variable	Odds ratio (95% confidence interval)	P
**Beta-blocker**	11.34 (1.97; 65.38)	0.007
**Valvular disease**	2.18 (0.38; 12.41)	0.380

Supplementary Table 3. Primers sequence for realtime PCR.

**Primer name**	**Sequence**
Nkx2.5 FW	GGTGGAGCTGGAGAAGACAGA
Nkx2.5 RV	CGCCGCTCCAGTTCATAG
MHC FW	CAGAAGAAGAAGATGGATGC
MHC REV	CGCTGGTGTCCTGCTCCT
TGFBR2 FW	CTGCACATCGTCCTGTGG
TGFBR2 REV	GGAAACTTGACTGCACCGTT
COL1A1 FW	AAGAGGAAGGCCAAGTCGAG
COL1A1 REV	CACACGTCTCGGTCATGGTA
COL3A1 FW	CATGCCCTACTGGTCCTCAG
COL3A1 REV	ATAGCCTGCGAGTCCTCCTA
HPRT-1 FW	TCCTCCTCCTGAGCAGTCA
HPRT-1 REV	ACCCTTTCCAAATCCTCAGC
GAPDH FW	ACAGTCAGCCGCATCTTC
GAPDH REV	GCCCAATACGACCAAATCC
KDR FW	AAAGGGTGGAGGTGACTGAG
KDR REV	CGGTAGAAGCACTTGTAGGC
Cx43 FW	AGGAGTTCAATCACTTGGCG

Cx43 REV	GAGTTTGCCTAAGGCGCTC
GATA-4 FW	GTTTTTTCCCCTTTGATTTTTGATC
GATA-4 REV	AACGACGGCAACAACGATAAT
IL-6 FW	GGTACATCCTCGACGGCATCT
IL-6 REV	GTGCCTCTTTGCTGCTTTCAC
IL-8 FW	CTTGGCAGCCTTCCTGATTT
IL-8 REV	TTCTTTAGCACTCCTTGGCAAAA
SDF1 FW	CTCCTGGGGATGTGTAATGG
SDF1 REV	GCCTCCATGGCATACATAGG
EGF FW	GGATAGCCAACAAACACACT
EGF REV	GGCACGTGCAGTAATAGGAT
FGF FW	CCTGGGGAGAAAGCTAT
FGF REV	GCTTCACGGGTAACAG
VEGF FW	CTACCTCCACCATGCCAAGT
VEGF REV	CCACTTCGTGATGATTCTGC
TGFbeta1 FW	GCAGCACGTGGAGCTGTA
TGFbeta1 REV	CAGCCGGTTGCTGAGGTA

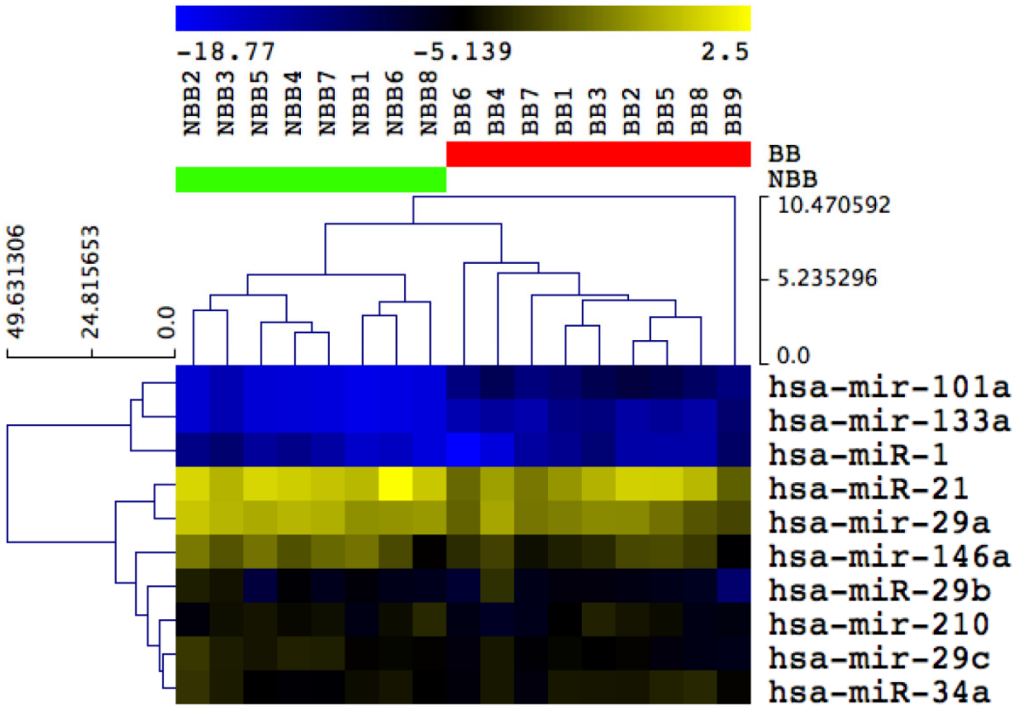

Supplementary Figure S1. Complete heatmap of microRNA expression profile between BB and NBB CPCs. Heatmap showing hierarchical clustering analysis for average euclidean distance of all miRNAs considered for the analysis.

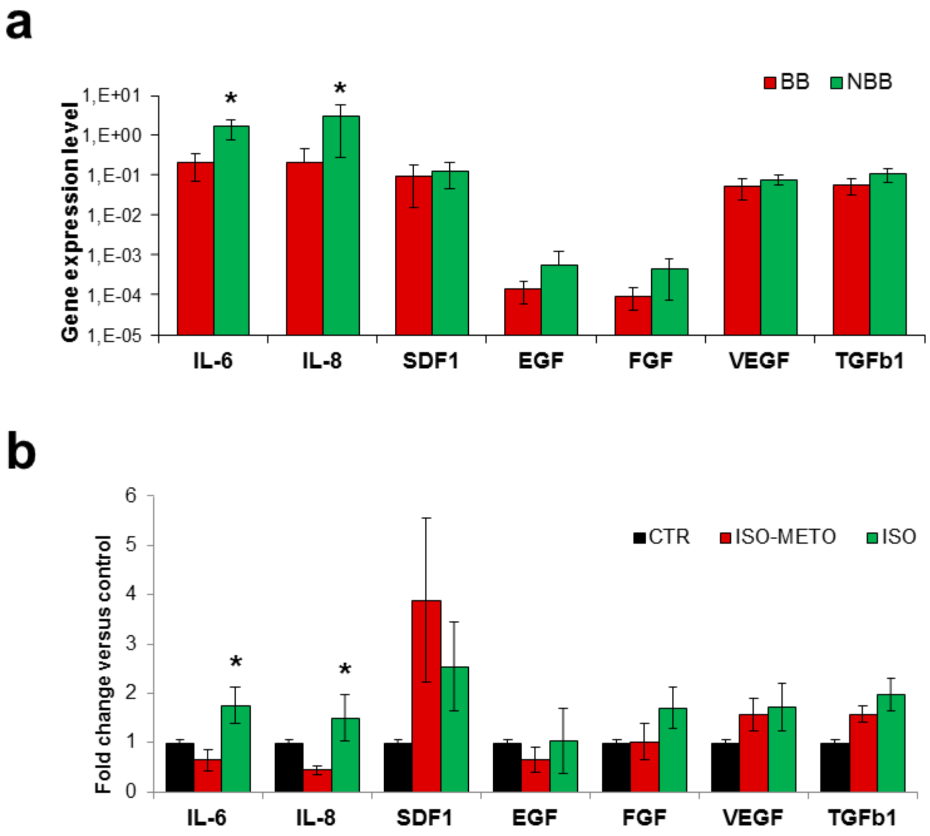

Supplementary Figure S2. Comparative interleukin and cytokine gene expression analyses.

Significantly higher gene expression levels for IL-6 and IL-8 (a) were detectable in non beta-blocker (NBB) versus beta-blocker (BB) CDCs (BB n=8, NBB n=4). Four-day isoproterenol (ISO) treatment in vitro (b) significantly increased IL-6 and IL-8 normalized gene expression levels in CDCs versus control (CTR), and this trend was reverted after subsequent metoprolol (METO) treatment for 4 more days (ISO-METO). (n=4). *= P<0.05.

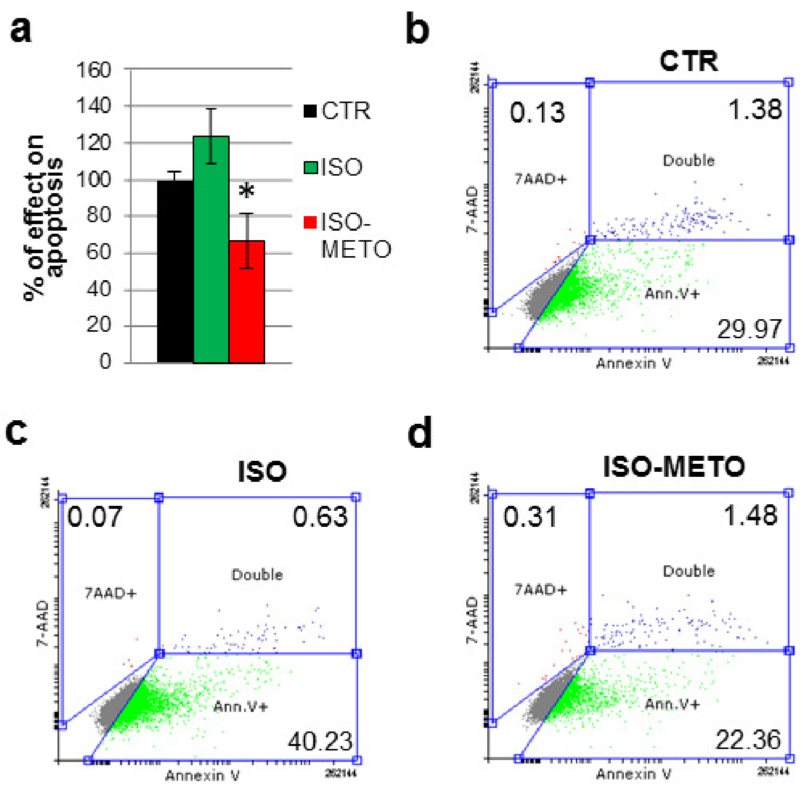

Supplementary Figure S3. Adrenergic pharmacological treatment in vitro affects apoptosis in CDCs. Four-day treatment with isoproterenol (ISO) was associated to a higher proportion of early-apoptotic AnnexinV+ CDCs versus control (CTR), while subsequent metoprolol (METO) treatment significantly reduced AnnexinV+ cells compared to both ISO and CTR cells (a). Representative cytofluorimetry dot plots are also shown (b-d). n=3. *= P<0.05.
